# Support via Technology: Living and Learning With Advancing Frontotemporal Degeneration

**DOI:** 10.1093/geroni/igaf122.016

**Published:** 2025-12-31

**Authors:** Allison Lindauer, Aimee Mooney

**Affiliations:** Oregon Health & Science University, Portland, Oregon, United States; Oregon Health & Science University, Portland, Oregon, United States

## Abstract

Support via Telehealth: Living and Learning with Advancing FTD (STELLA-FTD) is one of the few behavioral interventions in the US designed specifically for family care partners with all types of FTD (e.g., behavioral variant, primary progressive aphasia). Our NIA-funded study provides a group-based intervention that tests the efficacy of the ABC (Activator, Behavior, Consequences) approach, both in increasing care partner self-efficacy and reducing burden. The intervention teaches care partners about how to address behavioral symptoms by engaging rehabilitation specialists in their care journey. This aligns with the World Health Association’s mandate that dementia care include rehabilitation support. Along with testing the STELLA-FTD intervention, we are also testing the mechanism of action. In the early 2000’s Teri (2005) advanced the science of behavior analysis into the dementia field by making the ABC analytic approach accessible to care partners. This approach is well-known in the caregiving world, tested by scientists in Thailand, Brazil, Japan, and the US. These studies have shown positive results in reducing care partner burden, but none, to our knowledge, have isolated the essential components of the ABC analytic approach, and thus, the mechanism of action is not fully understood. Because of this, it is not known what the “essential ingredient” is in ABC-based interventions. This RCT will isolate the ABC approach in the context of a telehealth-based intervention. This will allow future studies to engage the known mechanism of action, potentially increasing the effectiveness of behavioral interventions for care partners.

